# Supplementary figures and images for: The PitA protein contributes to colistin susceptibility in Pseudomonas aeruginosa
Source: PLoS One. 2023 Oct 12;18(10):e0292818. doi: 10.1371/journal.pone.0292818 (PMC10569645; doi:10.1371/journal.pone.0292818)

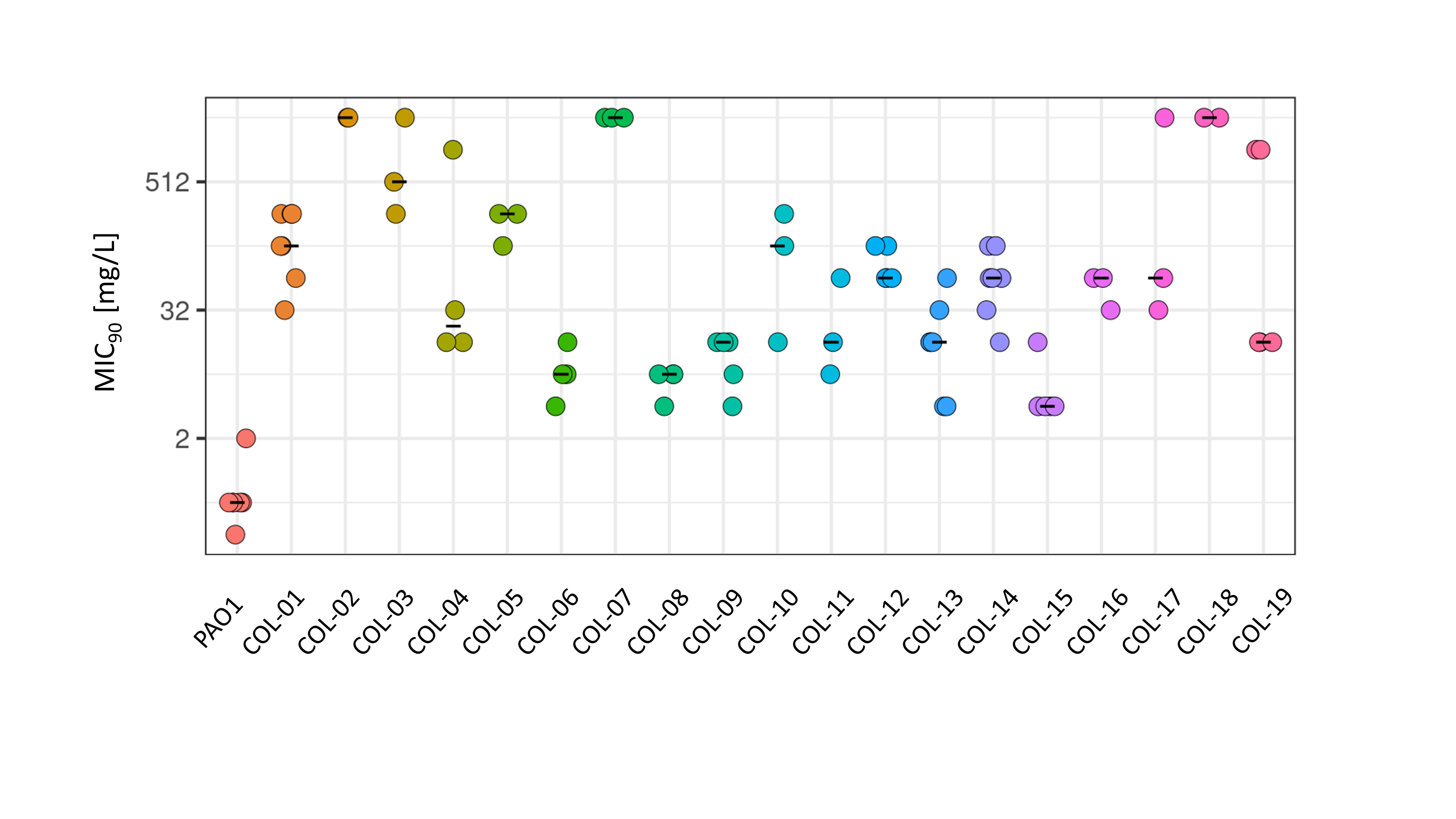

Supplement: S1 Fig — Biological replicates are displayed as individual data points, with black lines indicating the median values. (TIF) [file pone.0292818.s001.tif]

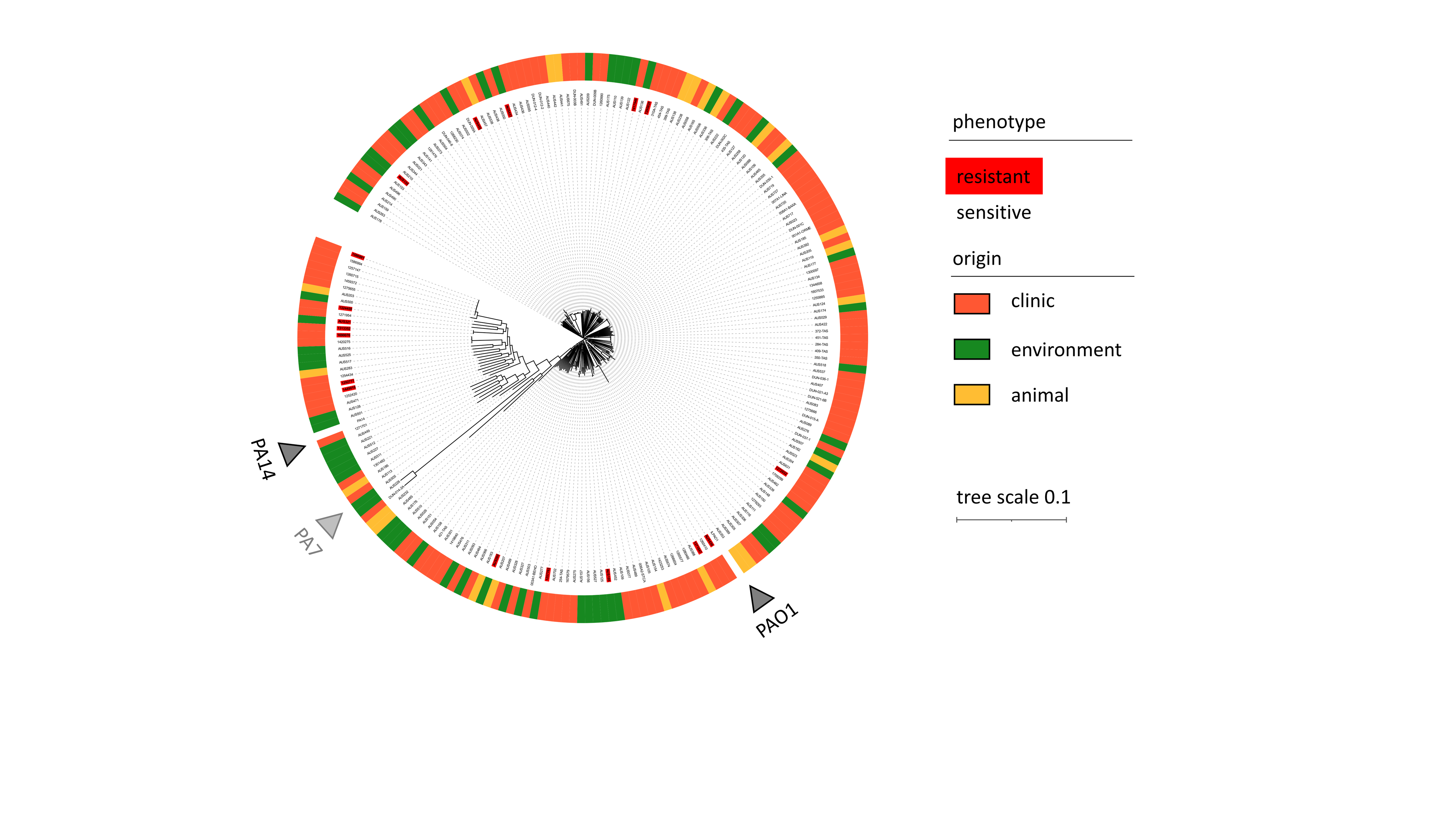

Supplement: S2 Fig — 214 P. aeruginosa isolates of clinical, environmental and animal origin (S4 Table in S1 File) were phylogenetically analyzed using parsnp and the tree was visualized using iTOL. Inner circle: colistin-resistant isolates are shown in red, with colistin-sensitive isolates in black. The widespread distribution of colistin-resistant isolates demonstrates that resistance has arisen on multiple occasions. Outer circle: source of isolate. The reference strains PAO1 and PA14 and the location of reference strain PA7 are included to visualize the variety of the isolates. (TIF) [file pone.0292818.s002.tif]

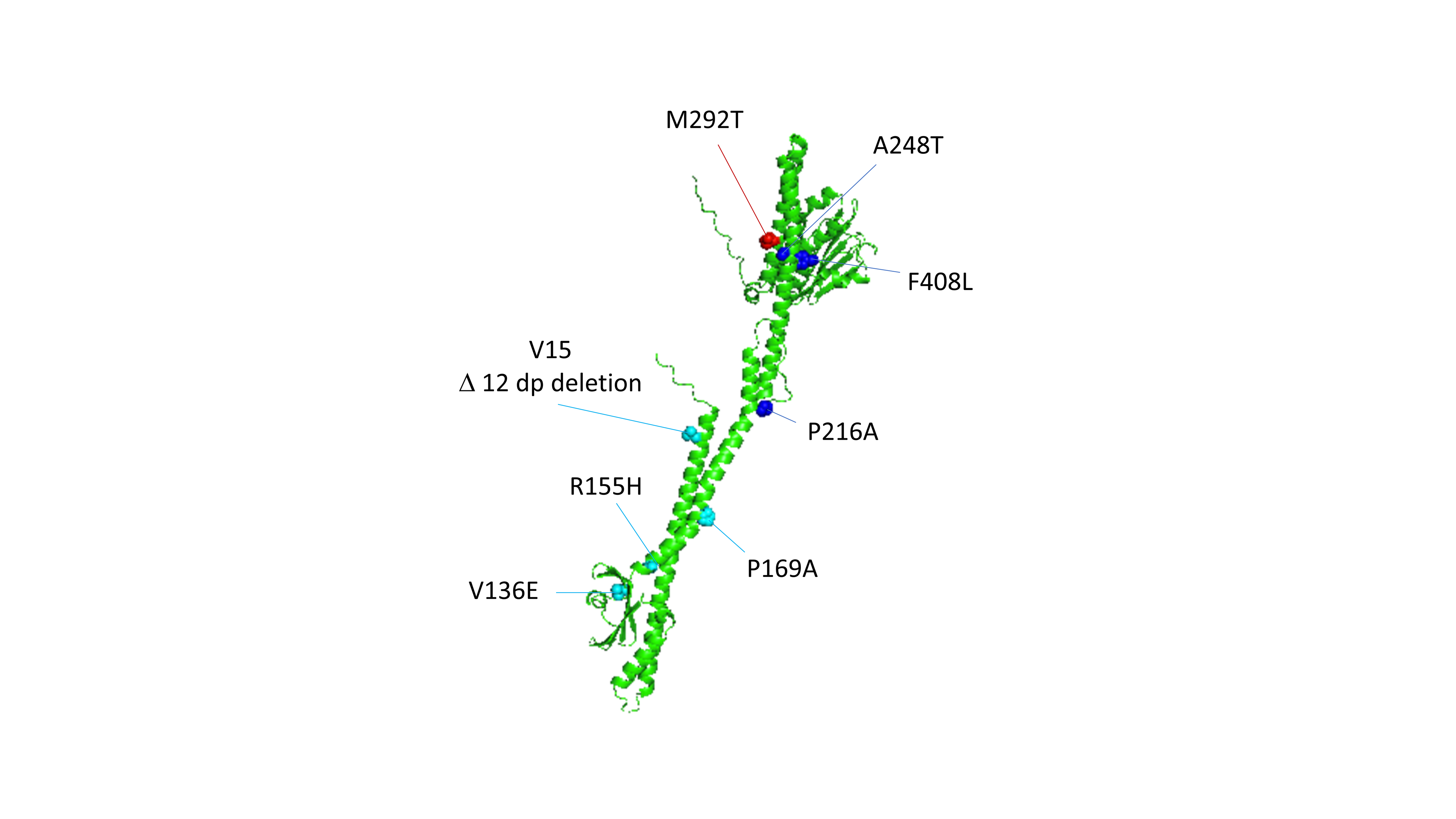

Supplement: S3 Fig — The predicted PmrB protein structure (UniProtKB Q9HV31) was visualized using PyMOL. The structure of the protein is shown in green, with changes in evolved mutants highlighted as spheres. The M292T mutation in red was engineered into PAO1, mutations in cyan were present in evolved mutants with only one mutation and mutations in blue were present in evolved mutants with multiple mutations. For the Δ12 bp deletion mutation the first affected amino acid in the protein was coloured. (TIF) [file pone.0292818.s003.tif]
