# Supplementary material for: The PitA protein contributes to colistin susceptibility in Pseudomonas aeruginosa
Source: PLoS One. 2023 Oct 12;18(10):e0292818. doi: 10.1371/journal.pone.0292818 (PMC10569645; doi:10.1371/journal.pone.0292818)
Supplement: Attachment — Submitted filename: Response to reviewers.docx [file pone.0292818.s005.docx]

**Response to reviewer comments: manuscript PONE-D-23-24646**

Reviewer and staff comments are shown in normal font, with our responses in italics. All references to line numbers in our responses refer to the numbering in the marked up revised manuscript.

**Response to reviewer comments: Reviewer #1.**

About the Table 3, it may be helpful addition to analyze the impact of different single gene mutations on other antibiotic resistance.

*We carried out this analysis. There was no relationship evident between the single gene mutations and resistance to antibiotics other than colistin. As this result was not informative, we have not described it in the manuscript.*

It seems phosphate deficiency may play a role in LPS modification. I would like to know if authors considered exploring the contribution of PitA to polymyxins susceptibility in a phosphate deficient environment ?

*This is an interesting suggestion, thank you. We have not explored the effects of phosphate deficiency on colistin susceptibility, which is beyond the aims and scope of this study. However we have now noted this in the discussion (lines 281-282) as a future research direction.*

**Changes to meet journal requirements:**

We note that you have provided funding information that is not currently declared in your Funding Statement. However, funding information should not appear in the Acknowledgments section or other areas of your manuscript. We will only publish funding information present in the Funding Statement section of the online submission form.

Please remove any funding-related text from the manuscript and let us know how you would like to update your Funding Statement. Currently, your Funding Statement reads as follows: 
 "This work was supported by a grant from the Health Research Council of New Zealand (17/372) (www.hrc.govt.nz) to IL. The funders had no role in study design, data collection and analysis, decision to publish, or preparation of the manuscript."

Please include your amended statements within your cover letter; we will change the online submission form on your behalf.

*We apologise for this error. We have now deleted the relevant sentence from the Acknowledgements section. We have included the new Funding Statement, using the wording recommended by the editorial staff, in our cover letter. Thank you in advance for changing the online submission form.*

In your Data Availability statement, you have not specified where the minimal data set underlying the results described in your manuscript can be found.

*We have incorporated underlying data into the supplementary tables.*

*Data availability:*

*Figures 1A: underlying data are in Table S8*

*Figure 1B: underlying data are in Table S1 and Table S9*

*Figure 2: underlying data are in Tables S10 and S11*

*Table 1: underlying data are in Table S2*

*Table 2: underlying data are in Table S4*

*Table 3: underlying data are in Table S12*

Please review your reference list to ensure that it is complete and correct.

*We have reviewed this list and it is complete and correct. There are no changes from the previous version of the manuscript.*
